# Assessing Knowledge and Barriers at the Primary Care Provider Level that Contribute to Disparities in Inflammatory Breast Cancer Diagnosis and Treatment

**DOI:** 10.21203/rs.3.rs-2302308/v1

**Published:** 2022-12-05

**Authors:** Gayathri Devi, Laura Fish, Alexandra Bennion, Gregory Sawin, Sarah Weaver, Anh Tran

**Affiliations:** Duke University School of Medicine; Duke University School of Medicine; Duke University School of Medicine; Duke University School of Medicine; Duke University School of Medicine; Duke University School of Medicine

**Keywords:** breast cancer, rare cancer, primary care provider, health disparity, CME

## Abstract

**Purpose:**

The purpose of this study was to evaluate knowledge gaps and barriers related to diagnosis and care of inflammatory breast cancer (IBC), a rare but most lethal breast cancer subtype, amongst Primary Care Providers (PCP) as they are often the first point of contact when patients notice initial symptoms.

**Methods:**

PCP participants within Duke University Health System, federally qualified health center, corporate employee health and community practices, nearby academic medical center, Duke physician assistant, and nurse leadership program alumni were first selected in a convenience sample (n=11) for semi-structured interviews (n=11). Based on these data, an online survey tool was developed and disseminated (n=78) to assess salient measures of IBC diagnosis, health disparity factors, referral and care coordination practices, COVID impact, and continued medical education (CME).

**Results:**

PCP reported access to care and knowledge gaps in symptom recognition (mean = 3.3, range 1–7) as major barriers. Only 31% reported ever suspecting IBC in a patient. PCP (n=49) responded being challenged with referral delays in diagnostic imaging. Additionally, since the COVID-19 pandemic started, 63% reported breast cancer referral delays, and 33% reported diagnosing less breast cancer. PCP stated interest in CME in their practice for improved diagnosis and patient care, which included online (53%), lunch time or other in-service training (33%), patient and provider-facing websites (32%).

**Conclusions:**

Challenges communicating rare cancer information, gaps in confidence in diagnosing IBC, and timely follow-up with patients and specialists underscores the need for developing PCP educational modules to improve guideline-concordant care.

## Introduction

In the United States, the NCI defines rare cancers as those which occur in fewer than 15/100,000 people each year, representing about 27% (400,000 Americans) of all US cancer diagnoses [1]. In Europe, the Surveillance of Rare Cancer in Europe (RARECARE) [9, 18] it is < 6/100,000 per year with 24% of all cancer diagnoses. The five-year survival rates for rare cancers are also lower than those for common cancers, accounting for a disproportionately higher rate (25%) of all cancer deaths [1].

There are around 200 forms of rare cancers, and they are generally understudied compared to common cancers, resulting in diagnostic criteria and standards of care similar to those for common cancers in that organ/type [34]. Breast cancer, the most common cancer in women worldwide, is one such example where recent improvements in treatment options have considerably increased survival outcomes [25]. However, inflammatory breast cancer (IBC) - a rare type representing only 1–6% of all breast cancer cases - constitutes 10% of all breast cancer deaths globally [2]. Inherent to rare diseases, barriers to timely IBC diagnosis include lack of awareness and mis- or late-diagnosis due to lack of a palpable mass [5, 6, 11] that could be detected by self-exam or mammogram. In addition, few IBC-specific clinical trials exist due to low patient numbers and biobanking – all leading to significant treatment delays and poor clinical outcomes. Currently, IBC patients receive a trimodal regimen of chemotherapy, surgery, and radiation, similarly as locally advanced breast cancers, but with reduced overall survival outcomes [3, 10, 15]. In addition, 30% of IBC patients present with metastatic disease, further underscoring the importance of a prompt diagnosis [35]. Most importantly, IBC is a NIH-designated cancer health disparity [26] with increased global incidence and mortality in minoritized and marginalized populations [20, 37, 38]. This is consistent with reports of distinct reproductive risk factors like younger age at first pregnancy, multiparity, and breastfeeding in IBC [16] [28, 30]. These epidemiological studies highlight the significance of primary care providers (PCP) including physicians, physician assistants, and nurse practitioners, who are often the first point of contact when patients begin to notice symptoms [31]. Although PCP play a key role in prevention and early detection of IBC, few studies have examined PCP knowledge and practices related to IBC. To fill this gap, we conducted qualitative interviews with PCP to better understand experiences with IBC and to develop a survey instrument to quantitatively measure PCP experience related to awareness, barriers, and facilitators of identification and treatment of IBC. In this article, we report the providers’ IBC knowledge, attitudes, structural practice barriers, and the need for educational strategies to improve diagnosis and care.

## Methods

### Study Design

We conducted formative mixed methods research to develop and implement the questionnaire in two phases (key informant qualitative interviews and an online quantitative survey) described below.

### Study Participants

For qualitative key informant interviews, 11 participants were identified through purposive sampling and included local PCP based at Duke University Health System (DUHS), a nearby federally qualified health center, and a corporate employee health practice. A subset of participants from the first phase reviewed the survey draft prior to dissemination in the second phase. For the online survey, participants were recruited (August 2021-March 2022) by a combination of both convenience and snowball sampling methods targeting PCP based within DUHS, a nearby academic medical center, local community practices, and alumni from two Duke-affiliated Advanced Practice Provider degree and leadership programs. Participant convenience sampling occurred via email announcement distributed to eligible provider membership within the target entities. A supplemental number of PCP were also contacted using a snowball sampling approach whereby the study team encouraged eligible participants to share the study team email with other PCP acquaintances. Interested prospective participants could then contact the project for more information or directly respond to the online link to complete the survey anonymously. Because emails were sent to practice leaders who then internally disseminated the survey, based on the practice size information, we estimate a total of around 300 PCP were sent the survey link. This study was approved by the Duke University Institutional Review Board.

### Qualitative interviews

In the first phase, we conducted interviews with key informants to explore PCP knowledge, attitudes, and practices related to IBC. Between August 2020 and April 2021, experienced qualitative interviewers conducted interviews with PCP via Zoom (Zoom Video Communications, Inc.; San Jose, CA). We created an interview guide, informed by our literature search, prior research, theories and frameworks [13, 14, 39, 44]. The interview questions focused on differentiating IBC from common breast cancers; IBC symptom recognition and diagnosis; health disparity issues at the patient, provider, and community level; explaining IBC to patients; referral practices; and connecting with specialized clinical centers (Online Resource 1). Interviews lasted approximately 30 minutes and were audio recorded prior to transcription by a research assistant. Next, we pre-tested the first draft of the questionnaire using a cognitive interview process, via a telephone call, with five additional providers from the same set of clinics. The core goal of cognitive interviews is to refine a survey instrument, find barriers to identifying the type of information desired, and reduce response errors [43].

### Fielded PCP questionnaire

Based on the key informant and cognitive interview data, we finalized and administered an online survey, via the secure web application, REDCap [23, 24], to assess PCP knowledge, attitudes, and practices for identifying and treating IBC. This quantitative survey (Online Resource 2) aimed to assess: 1. information needs 2. referral and care coordination patterns and 3. potential knowledge gaps and care coordination issues among PCP. Participant demographics, role, type of practice, years of experience, and average number of patients seen were also collected in the questionnaire.

## Data Analysis

### Qualitative

We met weekly throughout data collection and analysis to discuss the process and emergent themes, and key analytic decisions were documented. Initial key informant interview recordings were analyzed by researchers using an inductive content analysis to identify themes related to barriers and facilitators to identifying and treating IBC. Themes identified from key informant interviews guided development of the questionnaire. Next, we used a rapid analytic approach to summarize the cognitive interviews [22, 27, 40], as the reduced timeframe of rapid methods tends to be more deductive and explanatory than inductive and exploratory. Researchers summarized each interview using a deductive template based on the interview guide, and analyzed template data to identify major themes in each category.

### Quantitative

Descriptive statistics and figures were generated from survey data using GraphPad Prism version 9.3.1 for macOS (GraphPad Software, La Jolla, California, USA, www.graphpad.com). Categorical variables were summarized in frequencies and percentages.

## Results

### PCP Survey Development (Qualitative)

For the survey development phase, key informant and cognitive interviews were conducted with a convenience sample of 11 providers. Table 1 describes participant characteristics. Qualitative analysis allowed for identification of specific barriers and facilitators in each category, as discussed below, toward questionnaire refinement and finalization of the survey instrument. *Online Resource 3* includes representative quotes related to the following subthemes.

### Barriers in differentiating a rare subtype amongst other breast cancers

Overall, providers acknowledged that IBC is a rare cancer, and most had not seen patients with IBC in their practice. They reported a general awareness of unique symptoms of IBC but recognized that IBC is not likely to be part of typical differential diagnosis when patients present with mastitis or skin changes. In particular, respondents mentioned the importance of monitoring skin changes in the breast when patient complaints include pain and itching. In such cases, providers reported prescribing antibiotics and recommending a return visit in 7–10 days. Some providers reported that they would also refer the patient for a mammogram.

### Barriers related to cancer health disparity

#### Race-related

Providers were aware of racial/ethnic disparities in cancer diagnosis and treatment. Although none of the participants indicated direct experience with racial/ethnic disparities with IBC, most believed that factors related to other health disparities are likely similar with IBC. These include systemic racism, medical mistrust, access to care, socio-cultural constructs of health, stress, and comorbidities.

#### Rural-Urban divide

PCP responses included distance and transportation to clinics as major barriers to cancer diagnosis and treatment in general, and that distance to treatment locations likely contributes to observed disparities in rural areas. Sociocultural factors of health and provider shortages in rural areas can make the barrier of distance exponentially worse, contributing to mis- or late diagnosis and treatment delays.

#### Socioeconomic status (SES)

Participants described a link between SES and education, which may impact health literacy. Patients may delay care because they may not recognize signs and symptoms. Further, inability to pay for care can lead to patients ignoring signs and symptoms.

##### Knowledge Gaps regarding Standard of Care:

Providers stated they were not familiar with the epidemiology of IBC; they lacked understanding of the impact of age and that IBC incidence is higher in younger premenopausal women. General sentiments included: mammograms are probably the appropriate diagnostic test for IBC; acknowledgement that older women are less likely to have screening mammograms; and older patients likely have fewer health insurance barriers to accessing care due to Medicare coverage. One respondent encouraged older women to continue breast self-exams and include a yearly clinical breast exam.

#### Gender

One provider mentioned potential barriers to breast health promotion for transgender individuals, commenting, “Sexuality, especially for transgender patients, screening, how comfortable the provider is bringing up breast issues, breast exams, mammograms” may be limiting. This discomfort in providers may result in differential care, with fewer breast exams and screenings for transgender patients.

### IBC care: Barriers and facilitators

Providers discussed access to care as the single most important barrier to treating IBC and breast cancer in general. Issues like timely referrals, cost of care/lack of insurance, competing roles (caregiver or childcare) and transportation were mentioned. Providers identified the need for better access to specialists for consultation for rare conditions such as IBC. One provider suggested that partnership with cancer centers with expertise in rare cancers like IBC would be beneficial to improve relationship with PCP. All providers mentioned that they would submit a referral to a larger breast center in the local area and request a consultation with a provider there. However, participants noted some confusion regarding where to refer patients and what might be covered by patient insurance. Most reported that they would refer patients to radiology for a mammogram, with referral location dependent on insurance type.

Low health literacy and lack of trust in the health care system were also identified as potential barriers to IBC patient care. Providers mentioned the importance of supporting the patient’s follow-up with referrals to treat breast cancer. Providers reported that it is difficult to have all the information about a rare disease upfront during a patient visit, given the many aspects of care they must cover in the limited 20-minute appointment per patient. This is further compounded by the high volume of patients seen in a day, which can negatively impact their ability to recognize, educate, and refer patients for a rare condition such as IBC: “*Tyranny of the urgent and the expectation that I’m supposed to see 10–12 people in a half day. How deep can I get?*” (Online Resource 3).

### PCP Survey Dissemination (Quantitative)

**Figure 1:**
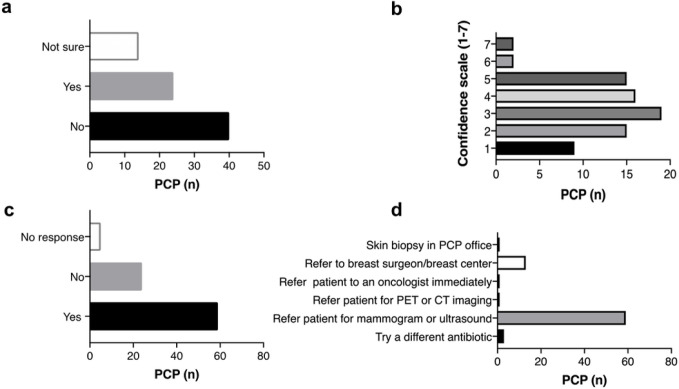
Barriers identified by Primary Care Providers (PA, NP, DO, MD) **a.** familiarity in recognizing IBC symptoms; **b.** level of confidence in diagnosing and treating IBC; **c.** experience related to referral delays; **d.** preferred follow-up care of patients suspected to have IBC.

Surveys sent out as an online link via email were completed by 78 PCP based within DUHS and local community practices in North Carolina. Table 2 describes respondent characteristics. The majority of respondents were primary care physicians (n = 49, 62.8%), and the remaining respondents included physician assistants (n = 21, 26.9%) and nurse practitioners/certified nurse midwives (n = 7, 9.0%). Although a majority of survey participants reported a high patient load (39.7% seeing > 40 patients/week), 65.3% estimated diagnosing < 5 common types of breast cancers among their patients per year. 30.8% of providers had ever suspected IBC in a patient, 51.2% had not, and 17.9% were unsure (Fig. 1a). Overall, PCP were only moderately confident in their ability to recognize IBC (mean = 3.3, range 1–7) (Fig. 1b).

To identify provider knowledge of the hallmarks of IBC, providers were presented with common clinical presentations of IBC (question 17; Online Resource 2). Responses revealed a gap in ability to correctly differentiate IBC from other breast cancers -- a result of high clinical relevance, with 44.2% of PCP respondents selected palpable breast mass as an IBC symptom. Other specific IBC symptoms of inverted nipple and nipple discharge (other than breast milk) were correctly identified by 69.2% and 57.1% of PCP respectively.

To assess steps taken when IBC is suspected, providers were presented with a hypothetical case describing a woman with left breast pain and inflammation, whose PCP prescribed antibiotics for a potential skin infection or mastitis (question 18; Online Resource 2). When asked what the appropriate follow-up care for this woman would be, should the condition not resolve with antibiotics, 75.6% responded that they would refer the patient for breast imaging (e.g., ultrasound, mammogram), 16.7% would refer the patient to a breast surgeon/breast center, 3.8% would try a different antibiotic, 1.3% would refer the patient for a PET scan/PT scan, 1.3% would refer the patient to an oncologist, and 1.3% would do a skin biopsy in the PCP office (Fig. 1d). Collectively, these data suggest gaps in clinical knowledge regarding the diagnostic hallmarks of IBC and the appropriate treatment for suspected IBC.

### COVID-19 Impacts and Telemedicine

Providers were asked about how the COVID-19 pandemic impacted screening and diagnosis of breast cancer among their patients, 33.3% (n = 26) of PCP reported lower breast cancer diagnoses than pre-COVID-19, 46.2% (n = 36) reported having made the same number of breast cancer diagnoses, and 11.5% (n = 9) indicated a higher number of breast cancer diagnoses in their care (Table 3). PCP also reported delays in breast cancer referrals since March 2020, with 62.8% (n = 49) of PCP reporting referral delays (Fig. 1c). Related to use of telemedicine, 28.2% (n = 22) of PCP responded conducting 11–20% of their visits via telemedicine (Table 3).

### Educational Strategies for PCP

Providers suggested that educational programs are needed to better understand IBC signs and symptoms and raise awareness. When asked what methods providers would find most helpful to learn more about diagnosing and caring for patients with IBC, the top three modes of preferred education were via online CME options (53%); lunch-time or other in-service training (33%); and websites for patients and providers (32%). In the qualitative interview phase, it was identified that partnering with the Duke IBC Consortium could be beneficial to learning about IBC. However, at the time of the survey, only 7.7% of providers were familiar with this entity. Some providers also suggested patient education in parallel is critical to helping women understand that skin changes in their breasts may indicate this rare cancer, along with peer coaching from women who have been treated for IBC. They reiterated the urgency to communicate, given the severity of the disease: *“… want to make sure it is not something serious like breast cancer, usually think of breast cancer as lump in the breast that gets bigger over time, but sometime can present in different ways like changes in the skin, want to do an additional evaluation just to make sure”* (quote #22, Online Resource 3). Several providers stated that it is important to discuss any skin changes and the possibility of cancer. Other responses included the need for a care plan that includes a core set of principles for diagnosing IBC, such as treatment algorithm, side effects, changes in surveillance, and plan over time, as well as provider training on how to deliver bad news to patients in a clear, concise manner. Ultimately, it is important to learn how to give information in a way that empowers or motivates the patients.

## Discussion

We succeeded in developing a survey instrument to assess PCP knowledge gaps and barriers to timely diagnosis and care of IBC patients. The responses collected post-survey dissemination revealed that although PCP are an essential part of the interprofessional approach to diagnosing and managing cancer patients, they lack knowledge of IBC symptoms, are uncertain regarding standardized IBC screening and treatment plans, and desire improved collaboration with cancer specialists. These three significant factors impact timely IBC diagnosis and treatment. Furthermore, participants indicated a desire to develop PCP-targeted IBC educational tools. To our knowledge, this is the first mixed methods study developed to identify the needs of primary care providers to effectively diagnose and treat IBC in the United States. The findings are significant and confirm a key theme that arose from an interactive community engagement session at an IBC national meeting with diverse stakeholder attendees (e.g., patients, clinicians, advocates, government, academic, and other health professionals and community members) to address critical needs in IBC clinical care and outreach [12]. In that national conversation, lack of education at the primary care provider level was identified as the primary factor contributing to disparity in diagnosis, care, and referral practices [13].

To address the difficulties of IBC symptom recognition among PCP, a clinical intervention of interest is the development of a clinical decision support system (CDSS) embedded within the electronic health record (EHR). CDSS are intended to improve healthcare delivery by enhancing medical decisions with targeted clinical knowledge, patient information, and other health information [32]. In the context of IBC, CDSS could be strategically used to aid in symptom recognition and diagnosis, imaging recommendation, and referral support. For example, PCP could input a patient’s symptoms and clinical observations into the CDSS and retrieve an output of a list of possible or probable diagnoses, including IBC. This output could also include a list of IBC specialists. This could be of immense value as IBC is associated with reproductive risk factors and mammary gland involution during post-pregnancy and lactation periods and, in general, a delay in cancer detection of up to 15 months has been reported in pregnant or breastfeeding women, leading to a 2.5-fold higher risk for diagnosis at an advanced stage [17].

Aside from symptom recognition, CDSS can aid PCP in ordering the most appropriate imaging, reminding them of best practice guidelines, and providing a list of specialists for referral [19]. Currently available data suggest that CDSS can have a positive impact on the quality of cancer care delivery [33] with the goal of being embedded directly into the EMR to avoid workflow disruption [42].

In addition, knowledge gaps among PCP regarding symptom recognition and management of suspected IBC highlight the need for the development of enhanced educational opportunities. PCP reported a preference to take online CME modules to improve differential diagnosis and their ability to educate their patient population. Recent literature has shown that CME is an effective mechanism to contribute to knowledge gain among primary care providers in the United States [21]. Specific to IBC, two studies report the benefits of CME programs employed in Egypt and Tunisia [39] and in Pakistan [41] toward improving IBC knowledge, early detection, and referral of IBC cases.

Breast cancer-screening guidelines in the US and Europe are increasingly recommending that a risk assessment for breast cancer be performed at the PCP level [7, 29]. Our survey instrument could be valuable in assessing deficiencies even before developing specific educational modules, a crucial step for effective learning among PCP [4] in cancer diagnosis, treatment, and survivorship care [8, 36]. In the future, it would be valuable to evaluate a pilot IBC CME program among PCP based on pre- and post-CME surveys of knowledge. It is also important to note that classic textbook images that do not capture the range of presenting signs and symptoms across skin tones may contribute to missed diagnoses in patients with atypical presentations. Thus, it is imperative to include diverse clinical presentations of IBC. We should also identify opportunities to integrate IBC education modules into existing clinical curriculums for PCP trainees (e.g., MD, PA, nursing). There is no reason to wait until PCP trainees enter into the clinical practice realm to enrich their understanding of IBC and how to better navigate care coordination, especially since participants in this study reported that with the advent of the COVID-19 pandemic, a significant proportion have experienced breast cancer referral delays, and are diagnosing less breast cancer. Following the development of IBC CME modules, future work is also needed to evaluate the efficacy of online CME among PCP. In conclusion, IBC is understudied and associated with a lack of care concordant guidelines, and the involvement of PCP from definition of diagnosis to quality monitoring has the potential to improve patient survival, and quality of life and to improve health equity. The survey instrument tested here has the potential to serve as a blueprint to design, implement, and evaluate interventions to support PCP in diagnosing and managing IBC and may be expanded to include other rare cancers.

## Figures and Tables

**Table 1:** Cognitive Interview Cohort Characteristics (n = 11).

Characteristic	Categories	% (n)
**Gender**	Male	36.4 (4)
	Female	63.6 (7)
**Role**	Nurse Practitioner/Certified Nurse Midwife	27.3 (3)
	Physician Assistant	27.3 (3)
	Physician	45.5 (5)
**Specialty**	Internal Medicine	9.0 (1)
	Family Medicine	91.0 (10)
**Number of Years Practicing Medicine**	5 to 9	27.3 (3)
	10 to 14	9.0 (1)
	15 to 19	9.0 (1)
	20 or more	54.5 (6)

**Table 2:** Online Survey Cohort Characteristics (n = 78).

Characteristic	Categories	% (n)
**Gender**	Male	17.9 (14)
	Female	79.5 (62)
	Prefer not to answer	2.6 (2)
**Hispanic or Latino/Latinx**	No	91.0 (71)
	Yes	7.7 (6)
**Race**	Asian	6.4 (5)
	Native Hawaiian/Pacific Islander	1.3 (1)
	Black or African American	12.8 (10)
	White	67.9 (53)
	More than one race	2.6 (2)
	Other	1.3 (1)
	Prefer not to answer	7.7 (6)
**Role**	Nurse Practitioner/Certified Nurse Midwife	9.0 (7)
	Physician Assistant	26.9 (21)
	Physician	62.8 (49)
	Other	1.3 (1)
**Specialty**	Internal Medicine	47.4 (37)
	Family Medicine	46.2 (36)
	Obstetrics/Gynecology	2.6 (2)
	Other	3.8 (3)
**Number of Years Practicing Medicine**	< 5	17.2 (5)
	5 to 9	17.2 (5)
	10 to 14	20.7 (6)
	15 to 19	17.2 (5)
	20 or more	27.6 (8)
**Main Practice Setting**	Individual practice	5.1 (4)
	Group practice	23.1 (18)
	Hospital	17.9 (14)
	Academic medical center	65.4 (51)
	FQHC or FQHC like setting	5.1 (4)
	Employer-based clinic	7.7 (6)
	Other	2.6 (2)
Number of Patients Seen Per Week	0 to 10	9.0 (7)
	11 to 20	9.0 (7)
	21 to 30	21.8 (17)
	31 to 40	19.2 (15)
	More than 40	39.7 (31)
	Prefer not to answer	1.3 (1)

**Table 3:** PCP Response to COVID-19 Related Impact on Breast Cancer Care (n = 78).

Characteristic	Categories	% (n)

**Change in the number of breast cancer diagnoses made by PCPs since COVID-19**	Same number of diagnoses	46.2 (36)
	Lower number of diagnoses	33.3 (26)
	Higher number of diagnoses	11.5 (9)
	Prefer not to answer	9.0 (7)

**Percentage of patient visits conducted remotely or via telemedicine since March 2020**	< 5%	26.9 (21)
	5–10%	24.4 (19)
	11–20%	28.2 (22)
	21–30%	6.4 (5)
	>30%	7.7 (6)
	Prefer not to answer	6.4 (5)

**Delays in referrals to diagnostic imaging for breast cancer since March 2020**	No, never postponed	30.8 (24)
	< 5% delayed	9.0 (7)
	5–10% delayed	21.8 (17)
	11–20% delayed	5.1 (4)
	21–30% delayed	2.6 (2)
	> 30% delayed	3.8 (3)
	Unsure	20.5 (16)
	Prefer not to answer	6.4 (5)

## Data Availability

The datasets generated during and/or analyzed during the current study are available from the corresponding author on reasonable request.
